# Twenty-five year trends in body mass index by education and income in Finland

**DOI:** 10.1186/1471-2458-12-936

**Published:** 2012-10-31

**Authors:** Ritva Prättälä, Risto Sippola, Marjaana Lahti-Koski, Mikko T Laaksonen, Tomi Mäkinen, Eva Roos

**Affiliations:** 1 National Institute for Health and Welfare, Helsinki, Finland; 2 Finnish Heart Association, Helsinki, Finland; 3 Hjelt Institute, Department of Public Health, University of Helsinki, Helsinki, Finland; 4 Folkhälsan Research Center, Department of Public Health, University of Helsinki, Helsinki, Finland

**Keywords:** Body mass index, 25-year time trends, Education, Income, Finnish men and women

## Abstract

**Background:**

The socioeconomic gradient in obesity and overweight is amply documented. However, the contribution of different socioeconomic indicators on trends of body mass index (BMI) over time is less well known. The aim of this study was to investigate the associations of education and income with (BMI) from the late 1970s to the early 2000s.

**Methods:**

Data were derived from nationwide cross-sectional health behaviour surveys carried out among Finns annually since 1978. This study comprises data from a 25-year period (1978–2002) that included 25 339 men and 25 330 women aged 25–64 years. BMI was based on self-reported weight and height. Education in years was obtained from the questionnaire and household income from the national tax register. In order to improve the comparability of the socioeconomic position measures, education and income were divided into gender-specific tertiles separately for each study year. Linear regression analysis was applied.

**Results:**

An increase in BMI was observed among men and women in all educational and income groups. In women, education and income were inversely associated with BMI. The magnitudes of the associations fluctuated but stayed statistically significant over time. Among the Finnish men, socioeconomic differences were more complicated. Educational differences were weaker than among the women and income differences varied according to educational level. At the turn of the century, the high income men in the lowest educational group had the highest BMI whereas the income pattern in the highest educational group was the opposite.

**Conclusion:**

No overall change in the socio-economic differences of BMI was observed in Finland between 1978 and 2002. However, the trends of BMI diverged in sub-groups of the studied population: the most prominent increase in BMI took place in high income men with low education and in low income men with high education. The results encourage further research on the pathways between income, education, living conditions and the increasing BMI.

## Background

In the developed world those with lower socioeconomic position have a higher body mass index (BMI) and are more likely to be overweight or obese than those with higher socioeconomic position [1,2]. The results obtained from Europe [3,4] are in line with results from other continents.

Education and income are among the most common socio-economic indicators used in studies on obesity and overweight. European trend studies suggest that educational differences in BMI have persisted during recent decades [5-8]. The European studies [5-8] have analysed educational differences only, while both education and income have been included in some North American and Canadian studies. The trends of educational and income-related differences in BMI were divergent in the USA in 1960–1980. Among men the educational gradient turned from positive to negative, among women the negative association between education and BMI became stronger. Income was positively associated with BMI among men but negatively among women [9,10]. Later North American studies observed parallel increases in BMI in all educational and income groups in 1986–2006 [11] and 1960–2008 [12] or did not find consistent changes in educational or income gradients (1970–2008) [13]. In Canada education and income related differently to BMI in 1978 and 2005. Education was inversely associated with BMI and there was no evidence of narrowing. The association was stronger for women than men. No clear association between income and BMI was observed for men whereas for women the association was inverse in 1978 but positive in 2005 [14]. The previous trend studies do not give a consistent picture of educational and income-related gradients of BMI. The associations of education and income with BMI seem to change over time and vary by populations and their subgroups.

European studies have analysed in cross-sectional settings the relative importance of several dimensions of socio-economic position as determinants of BMI. Educational level seems to have the most systematic association with BMI. Two studies compared variation in BMI or overweight in several European countries [3,15]. They showed that educational level was a stronger determinant of overweight than occupation or income. In particular, the association with income and overweight diminished after adjusting for education.

Education and income are both independent and interrelated indicators of socio-economic position: education enhances knowledge and non-material resources while income provides material resources. Educational qualifications contribute to later income. Educational level is more stable than income over the life course. To understand the interrelations between the socio-economic determinants of health it is not sufficient to search for just one statistically strongest socio-economic determinant [16]. The same can be applied to studies on socio-economic determinants of BMI. The previous studies have not paid attention to interactions between the determinants. The analysis of interactions between education and income over time could illuminate the origin of socio-economic variation in BMI and contribute to identifying population subgroups that are most vulnerable to weight gain.

Educational and income differences in BMI may change according to the economic situation of the study population. The economic situation changed greatly in Finland during the 1990s. After a period of growth in the late 1980s, the Finnish economy experienced a recession. Income inequalities started to increase and this trend has continued since. The increasing income equalities may have contributed to the increase observed in health inequalities [17,18]. According to a Finnish review, BMI has increased remarkably but the socioeconomic differences seem to have remained rather stable [19]. However, it could be assumed that during a period when the distributions of overweight, education and income have changed, the associations of education and income with BMI and the interactions between them might also have changed.

The overall aim of this study is to analyse the trends of socioeconomic differences in BMI among Finnish men and women from 1978 to 2002, during a period of increasing income and health inequalities. In order to analyse the importance of education and income, a specific emphasis will be put on a comparison of the two measures of socioeconomic position and on their possible overlap. We assume that changes in income-related differences in BMI are more obvious than the educational ones because the overall income-inequality became more prominent during the study period. Finally, we will cross classify education and income at different time periods in order to identify the population subgroups that have been most vulnerable to weight gain.

The specific research questions were to examine whether:

1. education and household income were independently associated with BMI,

2. the associations of education and income remained stable over a 25-year period since 1978,

3. there existed interactions between education, household income and study period. That is, were the income differences in BMI similar in each educational group during the two decades?

This paper is based on cross-sectional surveys repeated annually among 15–64-year-old Finns since 1978. The survey data was complemented with register data on household income. We will first present the associations of educational level and income with BMI from 1978 to 2002, and then analyse whether income differences in BMI are stable and similar in each educational group.

## Methods

The data were derived from a series of repeated cross-sectional health behaviour surveys. A nationwide random sample from the Finnish population aged 15 to 64 years was drawn annually during the period 1978–2002, with some 5000 Finns receiving a mailed questionnaire each year. The response rate has varied between 86 and 75% among women and 83 and 62% among men. The rate has declined over time [20]. The data have been collected according to the general ethical rules applied in the National Institute for Health and Welfare (and previously by the precursor National Public Health Institute). Individual participants cannot be identified from the data and only authorised persons have access to the data. Register linkages were not possible after the 2002 questionnaire because of changes in the ethical rules.

Employed respondents aged over 25 years were included in this study (N=57 351). All cases of missing data on income (4731), education (852) and BMI (666) were excluded. The proportion of missing income data was high during the first study period (9.4% for women, 16% for men) but decreased systematically over time. During the last study period 4.4% of income data was missing among women, 5.0 % among men. The study comprises data for a 25-year period (1978–2002) relating to 25 339 men and 25 390 women.

Educational level refers to years spent in full-time education as reported in the questionnaire. To take into account the increase in the general level of education in Finland since 1978, we divided the educational years into gender-specific tertiles per study year. Consequently education was classified into three groups: low, intermediate and high education (Table 1).

**Table 1:** Characteristics of respondents by study periods

		**1978-1982**		**1983-1987**		**1988-1992**		**1993-1997**		**1998-2002**	
		**Women**	**Men**	**Women**	**Men**	**Women**	**Men**	**Women**	**Men**	**Women**	**Men**
**BMI**	**M**	**23,8**	**25,1**	**23,8**	**25,3**	**24,0**	**25,6**	**24,4**	**25,9**	**24,6**	**26,1**
	*SD*	*3,6*	*3,1*	*3,7*	*3,2*	*3,8*	*3,4*	*3,8*	*3,4*	*4,2*	*3,5*
	*N*	*5254*	*6519*	*4448*	*4278*	*5565*	*5341*	*4996*	*4506*	*5127*	*4695*
**Household income per comsumption unit**											
low	**M**	**3220**	**3010**	**5960**	**5600**	**9410**	**9120**	**10640**	**10000**	**12220**	**11920**
	*SD*	*1130*	*1080*	*2320*	*2260*	*2610*	*2640*	*2970*	*2880*	*3530*	*3570*
	*N*	*1740*	*2165*	*1482*	*1421*	*1857*	*1774*	*1664*	*1500*	*1701*	*1556*
intermediate	**M**	**5800**	**5530**	**9720**	**9300**	**15040**	**15000**	**17360**	**17160**	**20400**	**20660**
	*SD*	*630*	*640*	*2510*	*2610*	*1870*	*1880*	*1900*	*2200*	*2230*	*2420*
	*N*	*1759*	*2171*	*1483*	*1430*	*1856*	*1782*	*1664*	*1502*	*1712*	*1569*
high	**M**	**9340**	**9190**	**15380**	**14970**	**24430**	**24830**	**28090**	**29380**	**36730**	**38250**
	*SD*	*3080*	*2900*	*7540*	*6020*	*10380*	*10290*	*8893*	*11100*	*40330*	*31980*
	*N*	*1755*	*2183*	*1483*	*1427*	*1852*	*1785*	*1668*	*1504*	*1714*	*1570*
**Education (in years)**											
low	**M**	**7**	**7**	**7**	**7**	**8**	**8**	**9**	**8**	**9**	**9**
	*SD*	*1,28*	*1,37*	*1,12*	*1,07*	*1,04*	*1,00*	*1,18*	*1,22*	*1,31*	*1,14*
	*N*	*2091*	*2286*	*1420*	*1502*	*1420*	*1825*	*1676*	*1471*	*1682*	*1493*
intermediate	**M**	**10**	**9**	**10**	**10**	**11**	**11**	**12**	**11**	**13**	**12**
	*SD*	*0,72*	*0,82*	*1,02*	*0,78*	*1,04*	*0,90*	*1,04*	*0,97*	*1,14*	*1,00*
	*N*	*1509*	*2007*	*1561*	*1353*	*1561*	*1821*	*1680*	*1567*	*1747*	*1707*
high	**M**	**14**	**14**	**15**	**15**	**16**	**16**	**17**	**16**	**17**	**17**
	*SD*	*2,54*	*2,82*	*2,24*	*2,56*	*2,11*	*2,35*	*2,19*	*2,34*	*2,24*	*2,56*
	*N*	*1654*	*2226*	*1467*	*1423*	*1850*	*1695*	*1640*	*1468*	*1698*	*1495*
**Means (M) and standard deviations (SD)**											

The information on respondents’ income was linked to the data from the national tax register of Statistics Finland. The linkage to data from the year 1985 was not possible due to missing ID-numbers in the survey data, meaning that the survey year 1985 could not be included in the study. Income data for the period 1978–1983 were based on participants’ income in the year 1980 and for the period 1984–1986 from the year 1985. Thereafter, data were available for each study year. The average household income per consumption unit was calculated from the total household income (gross income without income transfers). Consumption units were calculated according to OECD guidelines (=ECD 1982), with the household’s first adult person receiving a weight of 1.0, the other adults receiving a weight of 0.7, and children a weight of 0.5. Income was divided into tertiles for the analyses, representing low, intermediate and high income groups, which were calculated separately per study year and gender to represent the relative change in income (Table 1).

The tertiles of education and income were formed along the same principles in order to improve the comparability of the two indicators of socioeconomic position.

Body mass index (BMI) was calculated from self-reported height and weight (kg/m2). Persons whose BMI was 25 or over but below 30 were considered overweight, those with a BMI of 30 or more were classified as obese. The preliminary analyses were carried out by using both the prevalence of overweight and obesity as dichotomous variables and the mean BMI as a continuous variable. However, the trends and socioeconomic variations did not differ according to the outcome variable. As the main focus of this study was on the changes of BMI in socioeconomic groups and not on the proportions of obese or overweight persons, mean BMI was chosen for the final analyses.

The respondents were divided into 10-year- age groups 25–34, 35–44, 45–54 and 55–64. The research years were divided into five periods: 1978–1982, 1983–1987, 1988–1992, 1993–1997 and 1998–2002.

### Statistical methods

All statistical analyses were made using SAS version 9.1.3 (SAS Institute Inc., North Carolina, USA). The analyses were done separately for men and women to be able to identify differences in the patterns of variation between men and women. Multiple linear regression analysis was used to assess the effects of income and education on BMI. As BMI was not normally distributed among respondents, log transformation was used. All analyses were adjusted for age.

First, the effects of education and income on the BMI of men and women were analysed separately for each study period. Regression parameters and their p-values (p-values <.05 indicating statistical significance between the groups) were used to determine the educational and income differences in BMI. In addition, the magnitude of difference between the highest and lowest educational and income categories was calculated for each period from mean BMIs adjusted for age and for the other socio-economic variable. The magnitudes of differences were also presented as percentages. Second, the magnitude (difference between the first and last study period) of overall change between 1978 and 2002 was calculated for each income and educational group. When testing the statistical significance of changes for each period compared to the previous one linearity in mean log-BMI values was assumed. The changes were also calculated as percentages.

Finally, to see whether the income differences in BMI were similar in all educational level groups from 1978 to 2002, a time period variable was included into the model. In men the connection between BMI and time period was linear whereas in women it was non-linear. Time period was, therefore, treated as continuous in men and classified in women. Interaction terms and their p-values were used to determine whether different socioeconomic groups showed changes in BMI from one study period to another.

## Results

### Socioeconomic differences in BMI and their trends

Mean BMI was consistently higher among men than women, with the mean increasing among both genders between the first period (1978–1982) and the last (1998–2002) (Table 1).

Among women education had an independent effect on BMI during each study period: women having the lowest educational level had the highest BMI even after adjusting for income. Income had an independent effect on BMI, as well. The BMI in the highest income group was significantly lower than in the lowest, both before and after adjusting for education (Table 2). Educational level was associated with BMI also among the men. The educational differences were less systematic than among the women, as the intermediate educational group did not always differ from the highest educational group. Contrary to the women, income differences among the men were inconsistent and not statistically significant (Table 2). Among women the magnitude of the effects of education and income on BMI fluctuated between the study periods but remained statistically significant between 1978 and 2002. Among men the effect of education fluctuated accordingly (Table 2).

**Table 2:** The relative effect of education and household income to BMI in five different study periods in women and in men

**WOMEN**	**1978–1982**		**1983–1987**		**1988–1992**		**1993–1997**		**1998–2002**	
	**M**	**Adj.**	**M**	**Adj.**	**M**	**Adj.**	**M**	**Adj.**	**M**	**Adj.**
**Household income**										
low	24,1	23,9	24,0	23,9	24,0	23,9	24,4	24,3	24,6	24,5
intermediated	23,6	23,5	23,5	23,5	23,8	23,7	24,2	24,2	24,5	24,5
high	23,0	23,1	23,2	23,3	23,5	23,6	23,8	24,0	23,8	24,0
Difference high - low	−1,1***	−0,7***	−0,8***	−0,5***	−0,6***	−0,3**	−0,6***	−0,3**	−0,8***	−0,6***
Difference high - low **(%)**	−4,4	−3,1	−3,4	−2,3	−2,4	−1,3	−2,5	−1,4	−3,1	−2,3
**Education**										
low	24,1	24,0	24,2	24,2	24,4	24,3	24,8	24,7	24,8	24,7
intermediated	23,6	23,6	23,5	23,5	23,8	23,7	24,2	24,2	24,4	24,4
high	22,8	23,0	23,0	23,1	23,2	23,2	23,5	23,6	23,7	23,8
Difference high - low	−1,3***	−1,1***	−1,3***	−1,1***	−1,2***	−1,1***	−1,3***	−1,2***	−1,1***	−0,9***
Difference high - low **(%)**	−5,4	−4,5	−5,3	−4,6	−4,9	−4,5	−5,1	−4,8	−4,3	−3,7
**MEN**										
**Household income**										
low	25,0	24,9	25,1	25,0	25,4	25,3	25,7	25,6	25,9	25,8
intermediated	24,9	24,9	25,2	25,2	25,4	25,4	25,6	25,6	25,9	25,8
high	24,9	25,0	25,0	25,1	25,2	25,3	25,6	25,7	25,9	26,0
Difference high - low	−0,1	0,1	−0,1	0,1	−0,2*	0	−0,1	0,1	0,1	0,2
Difference high - low **(%)**	−0,5	0,4	−0,3	0,6	−0,8	0	−0,3	0,5	0,2	0,7
**Education**										
low	25,2	25,2	25,4	25,4	25,7	25,7	26,0	26,1	26,2	26,2
intermediated	25,1	25,1	25,2	25,2	25,4	25,4	25,5	25,5	25,7	25,7
high	24,5	24,5	24,7	24,7	24,9	24,9	25,3	25,3	25,8	25,7
Difference high - low	−0,7***	−0,7***	−0,7***	−0,8***	−0,8***	−0,8***	−0,7***	−0,7***	−0,4**	−0,5***
Difference high - low **(%)**	−2,8	−3	−2,9	−3	−3,2	−3,2	−2,7	−2,8	−1,6	−1,8

**M**=geometric mean adjusted for age, **Adj**.=geometric mean adjusted for age and the other SEP-variable, **Difference high – low**: p-values are calculated assuming linear change in mean log-BMI values for household income and education classes; p-value is the significance of change between ‘low’ category and ‘intermediated’ category and between ‘intermediated’ category and ‘high’ category. Numbers are the difference of BMI between ‘high’ category and ‘low’ category, **Difference high - low (%)**: difference high – low divided by mean BMI in ‘low’ category, **P-values:** *) <0.05, **) <0.01, ***) <0.001.

The mean BMI increased significantly (p<0.001) within each educational and income group from 1978 to 2002, with the sole exception concerning women with the lowest education. The percentage of change was 3.4% among women in the highest, 2.5% in the intermediate and 0.6% in the lowest educational group. The corresponding figures for income were 2.0% , 2,9 % and 1.5%. Among men the percentage of change was 4.0% in the highest, 1,5% in the intermediate and 2.8% in the lowest educational group. In regard to income the changes were 3.1%, 2,6% and 2,6% (data not shown).

### Changes in income differences of BMI within educational groups

**Figure 1:**
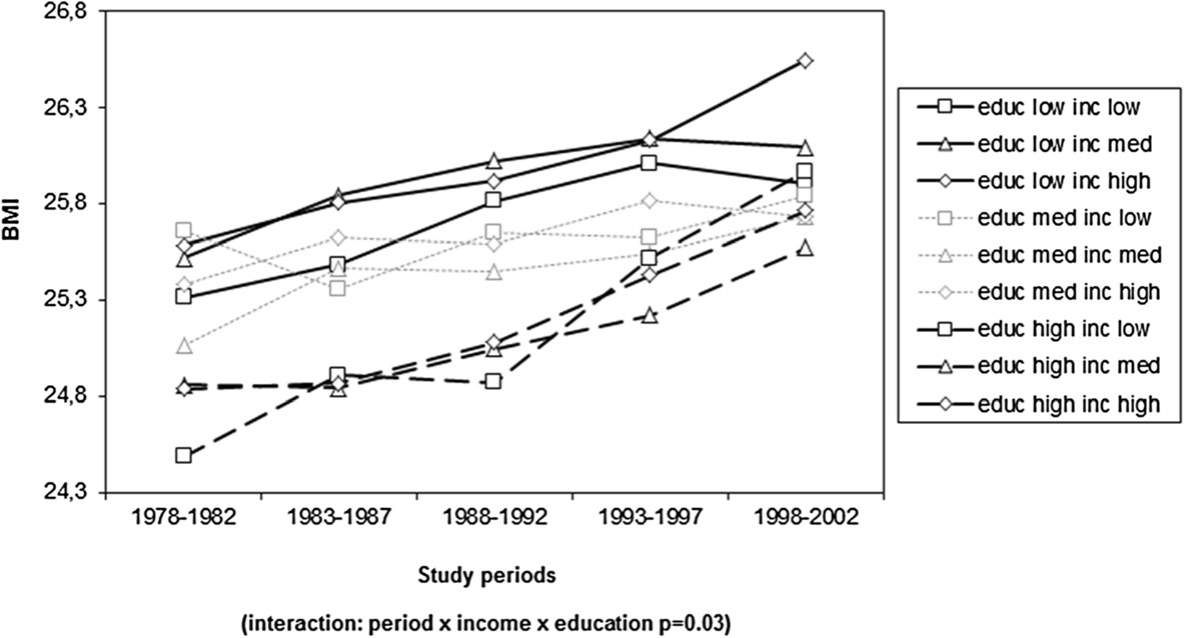
The shared effect of education and income on BMI in five study periods in men.

The shared associations of education and income demonstrate whether the income differences are stable in each educational group and vice versa. Figure 1 shows that the weight increase was greater among low educated men with high income and, on the other hand, among high educated men with low income. Among men a third grade interaction (p< 0.05) was observed between time period, education and income. This interaction suggests that income differences were not similar in each educational group. During the last time period, high income was associated with high BMI in the lowest educational group, whereas in the highest educational group, low-income men had the highest BMI (Figure 1). Among women a corresponding divergence in income-related patterns within the highest and lowest educational group was not observed (data not shown).

## Discussion

Our study showed a remarkable increase in BMI among 25–64-year-old Finns between 1978 and 2002. In women both education and income showed strong inverse associations with BMI. The magnitudes of the associations fluctuated but stayed statistically significant over time. Among the Finnish men, socioeconomic differences in BMI were more complicated. Educational differences were weaker than among the women and BMI did not seem to vary by income. However, income differences varied according to educational level. At the turn of the century, the high income men in the lowest educational group had the highest BMI whereas the income pattern in the highest educational group was the opposite. Our study did not show a systematic overall increase or decrease in the socio-economic differences between 1978 and 2002 but demonstrated diverging trends in the sub-groups of the studied population.

When interpreting the results, some methodological considerations need to be taken into account. Weight and height were self-assessed by the respondents. Self-assessed BMI is an underestimate of the measured BMI [21]. A recent Finnish study [22] that was based on measured BMIs from 1997 to 2002 demonstrated about 7% higher BMI in women and 4% higher BMI in men compared to our data. However, the increase was similar in both studies. Educational differences in measured BMI’s were also in line with our observations, especially among men. Among women the educational differences were smaller than in our study. The comparison of our results with the previous Finnish study suggests that women with higher education had a tendency to underestimate their body weight. Similar results have been reported from Australia [23]. As the focus of our study was not in absolute figures but on trends and variations between population groups, the bias caused by self-assessment barely disturbs our main conclusions.

Another methodological problem typical for surveys was also observed in our study. The response rate has decreased systematically over the twenty-year period, and more in low educated groups [20]. Thus, the socioeconomic differences are probably greater in the sample than among the respondents especially during the later study periods.

The use of household income data obtained from the national tax register was one of the strengths of our study. Unfortunately, the proportion of missing income data was high during the first study period. Subjects with missing income information had higher mean BMI (p<0.001) than the others during 1978–1982; later the difference was no more statistically significant. This suggests that our results overestimate the increase of BMI over time. As a consistent increase of BMI has been observed in other Finnish studies the possible overestimation may not be significant. Anyway, the results concerning the period 1978–1982 need to be interpreted with caution.

An advantage of a mailed questionnaire is its broad coverage and representativeness of the whole adult population in Finland. Another methodological strength of our study was the construction of comparable classifications of education and income for the analyses. The statistical power of the large number of respondents allowed stratified analyses of educational and income groups over a long time period.

Educational differences in BMI, overweight and obesity have been shown to exist in practically all Western countries during the last two decades [6,15,24-27]. Another common finding confirmed by our study is that educational differences are more systematic than those between income groups [3,9,10,15,28,29].

In line with previous studies, our study showed that socioeconomic patterns of BMI are more clear-cut among women than men and educational differences can be observed among both genders [30-32]. Roskam and Kunst [15] compared socioeconomic variation in European countries and analysed differences in the prevalence of overweight by educational attainment, occupational class and household income. Among men no occupational variations in overweight were observed after adjusting for the other socioeconomic variables. After controlling for education and occupation, household income was negatively associated with overweight in women but positively, although weakly, in men. Researchers have given several explanations for the larger socioeconomic variation among women. Women in higher socioeconomic position might be more concerned about their body shape and make more efforts to lose weight [30]; they also tend to have better employment status and less family demands than women in low socioeconomic position [4]. Among men, weight concerns and family demands might play a smaller role in all socioeconomic groups.

Our study did not show a consistent decrease or increase in educational differences of BMI. This finding is in line with previous Finnish (17, 20) and European (5–8) studies. We assumed that changes in income-related differences in BMI would be more obvious than educational ones because the overall income-inequality became more prominent during the study period. We did not observe an increasing income gradient across the studied subgroups. However, we identified diverging income trends among men in the highest and lowest educational group. In the 1990s, the upward-trend in BMI was the clearest among high-income men with low education and among low-income men with high education. By the end of the century the income gradient was negative in the lowest educational group but positive in the highest. These groups of men are in a contradictory situation, they belong to a low and high socioeconomic group at the same time. Maintenance of normal body weight seems to involve both the cultural resources associated with high education and the material resources associated with high income.

The low-educated high-income Finnish men have probably started with physically demanding jobs, without the motivation and skills to maintain an ideal weight as do the higher educational groups. High income has probably led to a decrease in physical activity and higher consumption of alcohol. A similar explanation does not apply to high-educated but low-income men. Their situation might reflect the consequences of economic downturn and the fact that they did not reach the income level of their high-educated peers. Picket et al. [33] and Wilkinson et al. [34] refer to psychosocial consequences of low socioeconomic position. The low socioeconomic position reduces people’s control over their life and work and has effects on health behaviours and body weight.

## Conclusions

During a period of economic downturn and an overall increase in income inequalities the mean BMI increased significantly but no systematic changes in educational or income-related differences were observed. Among Finnish men the association of income with BMI depended on educational level. At the turn of the century the income gradient was negative in the highest educational group but positive in the lowest. Our results encourage further research on the pathways between income, education, living conditions and the increasing BMI.

## Competing interests

There are no competing interests.

## Authors’ contributions

All authors participated in the specification of the study aims and in the interpretation of the data, commented on the manuscript versions, and approved the final version. RP planned the design of the study, participated in the interpretation of the data and wrote the first draft of the manuscript. RS was responsible of the statistical analyses and participated in the drafting of the manuscript. ML-K contributed to reviews on the previous studies of overweight and obesity. ML contributed to reviews on previous studies on health inequalities and construction of the socioeconomic variables. TM participated in the statistical analyses and provided expertise on the use of the data sets. ER contributed to reviews of previous studies on overweight and obesity.

## Pre-publication history

The pre-publication history for this paper can be accessed here:

http://www.biomedcentral.com/1471-2458/12/936/prepub
